# Seasonal variability of women’s dietary diversity and food supply: a cohort study in rural Burkina Faso

**DOI:** 10.1017/S1368980021004171

**Published:** 2022-09

**Authors:** Alissia Lourme-Ruiz, Christophe Kouamé Koffi, Denis Gautier, Dang Bahya-Batinda, Emmanuelle Bouquet, Sandrine Dury, Yves Martin-Prével, Mathilde Savy

**Affiliations:** 1 MoISA, Université de Montpellier, Cirad, Ciheam-IAMM, Inrae, Institut Agro, IRD, 911 Avenue Agropolis, Montpellier 34394, France; 2 Cirad, UMR MoISA, Montpellier, France; 3 Cirad, UPR Forêts et Sociétés, Université de Montpellier, Bobo-Dioulasso, Burkina Faso; 4 Université de Man, Côte d’Ivoire

**Keywords:** Dietary diversity, Seasonality, Food supply, Women, Farm households

## Abstract

**Objective:**

To investigate the seasonal variations of women’s dietary diversity (WDD) (items consumed and food supply) and its linkages with agriculture, market and wild resources.

**Design:**

A cohort of 300 women was followed-up over a year to investigate WDD and food sources (production, purchase or foraging). Monthly qualitative 24 h recalls allowed computing WDD Scores from a standard 10-food groups (FG) classification (WDDS-10). Associations between farm/women’s characteristics and WDDS-10 were investigated using multivariate mixed models including interaction terms factor*months.

**Setting:**

Tuy province, Burkina Faso.

**Participants:**

300 women of reproductive age.

**Results:**

Both dietary diversity and food sources were seasonal. The mean WDDS-10 was relatively stable from August to January (ranging from 3·1 to 3·5 FG) when farm production predominated. The WDDS-10 gradually increased from February, concomitantly with an increase in food purchases (onions, tomatoes, mangoes) and reached its highest levels (>4 FG) from March to June, when food purchases were still relatively high and when more women consumed foraged fruits (shea plums and wild grapes). Women living on farms owning > 3 plough oxen and different animal species had significantly higher WDDS-10 than others (+0·28 and +0·35 FG, respectively). Women who practiced off-farm activities also had higher WDDS-10 than those who did not (+0·21 FG, *P* < 0·05). Other factors, for example, the number of foraged edible species, provided advantages in terms of dietary diversity only during certain seasons (October – January, *P* for interaction < 0·01).

**Conclusions:**

Diversifying women’s diets throughout the year requires complementary interventions aimed at diversifying production, promoting foraging and increasing income-generating activities to enable food purchasing.

Dietary diversity is crucial for meeting micronutrient requirements and improving nutritional status^(1,2)^. In rural areas in low-income countries, dietary diversity is particularly low due to monotonous diets, primarily based on starchy staples served with a vegetable sauce and limited in nutrient-dense foods^(3,4)^. Within farm households, family members may theoretically acquire diversified foods from three main supply sources: (i) from a diversified production, consumed directly on the farm; (ii) from food purchases on markets, enabled through agricultural or non-agricultural incomes and (iii) from foraging by gathering, hunting or fishing. However, the results available from empirical studies are not unequivocal. Some authors observed that higher agricultural biodiversity was associated with better women’s^(5–8)^ and household^(9–12)^ dietary diversity. Other authors argued that market access had a greater effect on farm households’ dietary diversity than did farming diversity^(13,14)^. A critical review by Jones^(15)^ reported a positive but low association between dietary diversity and both agricultural biodiversity (including crops, animals, trees or plants) and purchases (farms’ access to market and the sale of part of the production); however, the author did not differentiate between studies using dietary diversity scores at the individual level and those at the household level even though these scores are meant to reflect different dietary dimensions, namely nutrient adequacy for the former, and economical access to food for the latter.

In reality, the supply sources of food consumed are too rarely compiled, and hence opportunities to disentangle linkages between agriculture, market and wild resources and dietary diversity are insufficient. Yet, we believe that the significant associations between dietary diversity and production/incomes observed in several studies, and often wrongly considered as causal, provide only a partial explanation of potential mechanisms since they did not examine the actual processes leading from agricultural production to dietary consumption^(15)^. Foods from more diversified agricultural production can be consumed on-farm but can also be sold to purchase other food or non-food products. Indeed, the nature of spending within households depends on individual preferences, and more or less money may be allocated to non-food purchases or ‘unhealthy’ products (e.g. alcohol, sweetened or fatty foods) by different people. In addition, food can be unequally shared between farm members.

Another challenge relates to the seasonality of diets in rural areas. Foods consumed and their supply sources may significantly vary across the year according to food or income availability. Yet most studies investigating dietary diversity rely on cross-sectional data, with or without repetitions across the year^(16–19)^, thus revealing only a partial snapshot of the situation and resulting in possible over- or underestimation of the dietary diversity of the annual diet. Several studies that looked at dietary diversity in rural Burkina Faso have called for more frequent intra-annual data collection to better understand seasonal variations^(5,6,16,17)^.

This study aims to contribute to a better understanding of the seasonality of dietary diversity and its linkages with agriculture, market and wild resources. We measured the dietary diversity among women of reproductive age living in farm households in rural Burkina Faso through monthly 24 h recalls over an entire year, and we investigated how food originating from agriculture, markets and nature contributed to dietary diversity across seasons. We chose to focus on women of reproductive age because they are key actors in-household diets and are usually in charge of meal provision, management and preparation. The ultimate goal of this work was to identify relevant paths to improve access to better dietary diversity for all women, all year long.

## Methods

### Setting

The study was carried out in the province of Tuy, in the region of Haut-Bassins in western Burkina Faso. The climate is tropical, with two main seasons: the dry season from November/December to April and the rainy season from May to October/November. The major economic activity is agriculture, mainly based on family farming and rainfed crops, especially cotton and maize. Rainfed crops are harvested between October and January, while horticultural crops are harvested from January to May. Agroforestry trees are present on 84 % of farm plots and almost all farms own farm animals^(20)^. Many households are also involved in off-farm activities such as trade, service or craft work. The presence of gold in the area offers jobs either at the industrial gold mining site in Houndé (the capital of Tuy) or numerous clandestine sites.

### Study design

We conducted a longitudinal survey in which women of reproductive age were followed-up every month over 1 year (October 2017 – September 2018). To be eligible for inclusion in the survey sample, women had to meet three criteria: being between 15 and 49 years old, living in a farming household and having no travel plans for the next 12 months.

All participants gave their written informed consent at the beginning of the study and confirmed their willingness to participate at each round of data collection.

#### Sampling

Participants were selected using a two-stage sampling method: 12 villages of the Tuy province were randomly selected from the administrative list with a probability proportional to their population size; then, 25 farms were randomly selected in each village using the most recent available national census^(21)^. Since this census was not recent (2006), the farms drawn were used as starting points from which we applied a random-route method to select the farms to be surveyed (*n* 300). This procedure ensured that households who arrived in the village after 2006 had a chance to be part of the sample. Within each farm, we surveyed the head of the farm for data related to the farm/household, as well as a woman between 15 and 49 years of age. If several women were eligible in the farm, the enumerator had to list all eligible women and randomly select one of them. In total, we collected data on 300 farm households and women during the first round in October 2017.

#### Data collection

Data were collected by a team of nine extensively trained surveyors who were managed by three field supervisors and the field coordinator. Each monthly round of surveys lasted 4 d. The one-to-one interviews were conducted at home using the CAPI technique (Survey CTO^(22)^), which ensured real-time data controls. The structured questionnaire solicited basic demographic and socio-economic characteristics of the women and their farm household and included an open-recall 24 h qualitative dietary recall, as well as information on food supply sources. Demographic and socio-economic characteristics were collected only once during the first round of surveys.

### Women’s dietary diversity

Women were asked to describe all the foods, drinks and snacks they had consumed the day prior to the survey (defined as a 24 h period beginning the moment they woke up), as well as all ingredients consumed for mixed dishes. Each food item consumed was entered using a pre-defined list of 247 items; surveyors could also enter new items if necessary. Food items were classified using the 10-food group classification recommended by the FAO for women of reproductive age^(23)^: (1) grains, white roots and tubers; (2) pulses (beans and peas); (3) nuts and seeds; (4) dairy; (5) flesh foods (meat, poultry and fish); (6) eggs; (7) dark green leafy vegetables; (8) vitamin A-rich fruits and vegetables; (9) other vegetables and (10) other fruits. For each woman, we computed a Women’s Dietary Diversity Score (WDDS-10), defined as the number of different food groups (FG: out of the 10 listed above) consumed over the last 24 h. We also computed the Minimum Dietary Diversity for Women (MDD-W), a dichotomous index which equals 1 if women consumed at least five of the ten FG, or zero otherwise. Women of reproductive age who reach the threshold of five FG are more likely to meet their nutrient needs^(24)^. As recommended^(23)^, foods consumed in small quantities (less than 15 g, roughly the equivalent of 1 tablespoon), such as *soumbala* (a fermented bean ball) or fish powder, were systematically classified in the ‘spices and condiments’ group and did not count in the score.

### Food supply sources

The food supply source was solicited for each food consumed by the women, and classified as: (i) on-farm production; (ii) purchases; (iii) foraging (gathering, hunting and fishing) or (iv) gifts. On-farm production included species consumed from women’s farms, but a percentage of the same species might also be sold (e.g. cereals, vegetables or peanuts). Foraging included species that were not deliberately planted but were still protected and fostered on the plots, like wild grape trees (*Lannea microcarpa*), red kapok trees (*Bombax costatum*), shea trees (*Vitellaria paradoxa*) or néré trees (*Parkia biglobosa*). The baobab tree was classified into different categories depending on the part consumed. Baobab leaves were considered *produced* because they come from trees planted in courtyards or home gardens and deliberately kept small through regular pruning, while the fruits (monkey bread) were considered *foraged* because they grew on unpruned trees.

### Other variables

Basic socio-economic characteristics (age, literacy, marital status, etc.) of women and farm heads were recorded in the first round 1, as well as structural information concerning households and farms (household size, possession of assets, farm size, horticultural plots, home garden, cotton production, etc.). Women’s individual financial resources and activities were recorded at the first round and updated twice during the follow-up, in May and August 2018. Other data were collected every month, such as ownership of livestock and plough oxen, production from orchard trees (yes/no), number of foraged edible species, off-farm income sources (cash transfers from family, gold mining, mill services, shop, restoration, sewing, beer brewing, masonry, carpentry, mechanics). Some variables were broken down into terciles or quartiles depending on the distribution in our sample. We considered citruses, cashews, guavas, papayas, mangoes, bananas and dates as orchard trees. The foraged edible species comprised shea tree (*Vitellaria paradoxa*), néré tree (*Parkia biglobosa*), red kapok (*Bombax costatum*), weda (*Saba senegalensis*), jackalberry (*Diospyros mespiliformis*), sweet detar (*Detarium microcarpum*), hog plum (*Ximenia americana*), Indian jujube (*Ziziphus mauritiana*), marula (*Sclerocarya birrea*), *subudga* (*Gardenia erubescens*), tamarind (*Tamarindus indica*), African grape (*Lannea microcarpa*), desert date (*Balanites aegyptiaca*), African custard apple (*Annona senegalensis*), moringa (*Moringa oleifera*), sickle senna (*Cassia tora*), false sesame (*Ceratotheca sesamoides*), kapok (*Ceiba pentandra*), African fan palm (*Borassus aethiopium)*, black plum (*Vitex doniana*), horn-fruited jute (*Corchorus tridens*), afzelia (*Afzelia africana*), black nightshade (*Solanum nigrum*), shona cabbage (*Cleome gynandra*) and green amaranth (*Amaranthus hybridus*).

### Statistical analysis

Data cleaning and statistical analyses were performed using Stata 15^(25)^. Basic characteristics and variables related to women’s diet and food supply sources (dietary diversity scores, food group consumption, food item consumption, food supply sources and the proportion of women who reached the MDD-W) were first described using means (sd) for continuous variables and percentages (95 % CI) for categorical variables.

Seasonal variations in dietary variables were examined using linear mixed models for continuous variables and logistic mixed models for categorical variables with the individuals as a random intercept to account for the non-independence of observations from the same individual. The models included three-level mixed effects because we nested individuals into their village (cluster) as a second random intercept. All analyses included these features.

Using the same three-level linear mixed model, we studied the associations between the WDDS-10 and the resources of women and their farms. We first tested bivariate associations between WDDS-10 and each variable. The variables either significantly associated with the WDDS-10 (*P* < 0·05) or conceptually related to access to diversified foods for farm households^(26,27)^ (such as women’s age, household size, farm size or home garden) were entered in the multivariate model one by one. From an exploratory perspective, we aimed at integrating the various dimensions that could explain dietary diversity amongst women^(27)^. However, some variables were removed from the model to avoid collinearity (such as number of crops, livestock, number of co-wives or farm head characteristics). Cotton production was included as a dichotomous variable because the quantities produced were associated with farm size. Interactions terms (months × variable) were added in the model to test the changes over time in associations between WDDS-10 and each factor.

## Results

### Sample characteristics

At round 1 (October 2017), women were 31 (±8) years old on average and only 14 % were literate (Table 1). Almost all of them were married and lived on farms headed by males. Household size averaged 7·4 (±5) members. Many women were involved in individual activities: 75 % managed an individual plots (18·7 % with horticultural crops), 33 % kept a home garden and 63·3 % of them gathered non-timber forest products. In addition, 76·3 % of women were engaged in off-farm activities. The average size of the family field was 8·5 (±9) hectares; 8·7 % of farms had a vegetable plot and 41·5 % a home garden. Almost all farms owned farm animals, particularly poultry, sheep and goats, and three-quarters owned plough oxen.

**Table 1:** Characteristics of the 300 women and their households at the first round of survey (October 2017; *n* 300, unless specified otherwise)

	%	95 % CI*
Women’s characteristics		
Age (years)		
Mean	31·5	
sd	8·4	
< 30	43·0 %	41, 44
30 to < 40	34·7 %	33, 36
40 and up	22·3 %	21, 23
Married (*v*. single, divorced or widowed)	96·5 %	94, 98
Literate (yes)	14·3 %	13, 15
Women’s resources		
Manages her own plots (yes)	75·0 %	74, 76
With horticultural crops (yes, *n* 225)	18·7 %	17, 20
Plots size (ha, *n* 225)		
Mean	0·5	
sd	0·8	
Manages home garden (yes)	33·0 %	31, 34
Manages non-timber forest products (NTFP) (yes)	63·3 %	62, 65
Off-farm activities (yes)	76·3 %	75, 78
Household characteristics		
Household size (number of people)		
Mean	7·4	
sd	5·3	
Farm heads characteristics		
Male-headed farm	97·3 %	97, 98
Age (years)		
Mean	43·5	
sd	13·0	
< 30	14·0 %	13, 15
to < 40	26·3 %	27, 28
40 and up	59·7 %	57, 59
Literate (yes)	28·7 %	28, 31
Household assets		
Possession of a radio (yes)	68·3 %	67, 70
Possession of a motorbike (yes)	60·7 %	59, 62
Possession of a moto-trailer (yes)	13·3 %	12, 14
Farm characteristics (managed by the farm head)		
Farm size (ha)		
Mean	8·5	
sd	8·8	
Cotton production (yes)	67·3 %	66, 69
Horticultural plot (yes)	8·7 %	8, 9
Home garden (yes)	41·3 %	40, 43
Orchard production (yes)	8·4 %	7, 8
Number of foraged edible species		
Mean	1·7	
sd	0·7	
Livestock ownership		
Farm animal (yes)	97·1 %	96, 97
Number of farm animals		
Mean	32·8	
sd	35·1	
Number of farm animals by species		
Cattle		
Mean	4·5	
sd	12·2	
Sheep and goat		
Mean	8·2	
sd	11·0	
Pig		
Mean	2·3	
sd	3·8	
Poultry		
Mean	16·7	
sd	19·6	
Pigeon		
Mean	0·7	
sd	4·1	
Plough oxen (yes)	72·0 %	71, 74
Number of plough oxen		
Mean	2·3	
sd	2·2	
Off-farm income sources		
No	39·3 %	37, 41
Yes, one activity	45·0 %	39, 42
Yes, two or more activities	15·7 %	19, 21

* For continuous variables, we present means and standard deviations; for categorical data, we present % and 95 % CI.

**Fig. 1:**
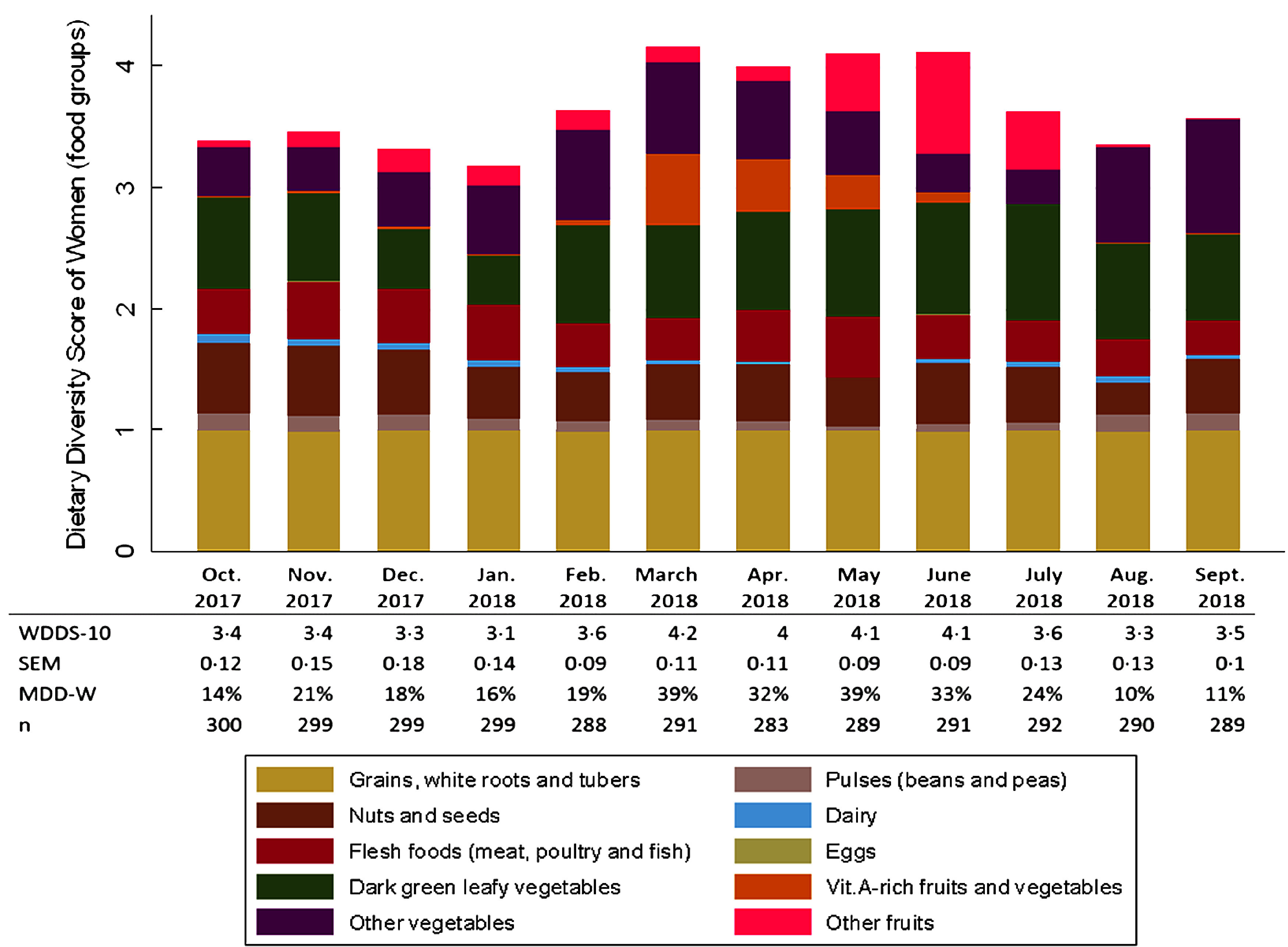
Seasonal variation in women’s dietary diversity and contribution of food groups to dietary diversity. WDDS-10, women dietary diversity score; MDD-W, minimum dietary diversity for women. Marginal predictive means (sem) for WDDS-10 and percentages for MDD-W are presented

Not all women were interviewed every month during the follow-up, due either to absence or lost-to-follow-up (defined as having missed at least six consecutive visits). However, response rates were high globally, the smallest number of women being 283 at round 7 (Fig. 1). Only four women were lost-to-follow-up (2 from February and 2 from April); however, the basic characteristics of these women were not significantly different than those of the other women in the sample.

### Seasonal variations in WDD

Over the year, the mean WDDS-10 varied from 3·1 FG in January to 4·2 FG in March, and the percentage of women reaching the MDD-W varied from 10 to 39 %. (Fig. 1). The mean WDDS-10 was higher from March to June (fluctuating between 4·0 and 4·2 FG) while the percentage of women reaching the MDD-W was between 32 and 39 % during this period. In contrast, during the rest of the year, the mean WDDS-10 varied between 3·1 and 3·6 FG and the percentage of women achieving the MDD-W fluctuated between 10 and 24 %.

Regarding food group consumption, we observed that staple foods were consumed by almost all women all year long (≥ 99 %); while contrarily, eggs and dairy products were marginally consumed (< 1 % and < 5 %, respectively) (Fig. 1 and see online Supplemental Table 1). The ‘nuts and seeds’ and ‘flesh foods’ groups were consumed on a regular basis over the year. ‘Nuts and seeds’ were, however, more consumed from September to December (> 50 %), in March (46 %) and in June–July (46–45 %). The consumption of flesh foods was highest in May (54 %) and lowest in August–September (28–29 %). The consumption of green leafy vegetables was relatively high over the year, and only declined in December–January (49 % and 40 %, respectively, *v*. ≥ 70 % for the rest of the year). In contrast, the consumption of ‘vitamin A-rich fruits and vegetables’, ‘other fruits’ and ‘other vegetables’ was highly seasonal. The consumption of vitamin A-rich fruits and vegetables reached 57·6 % in March, 44·5 % in April and 34·5 % in May, then dropped to 9·4 % in June and remained negligible the rest of the year. The proportion of women who consumed other vegetables ranged from 27·5 % (July) to 93·4 % (September), with greatest consumption between January and May, and between August and September. The consumption of other fruits was particularly high between May and July, peaking in June (83 %), and particularly low from August to September (1·7 % for both months) and in October (5·5 %).

### Food variety within food groups

**Fig. 2:**
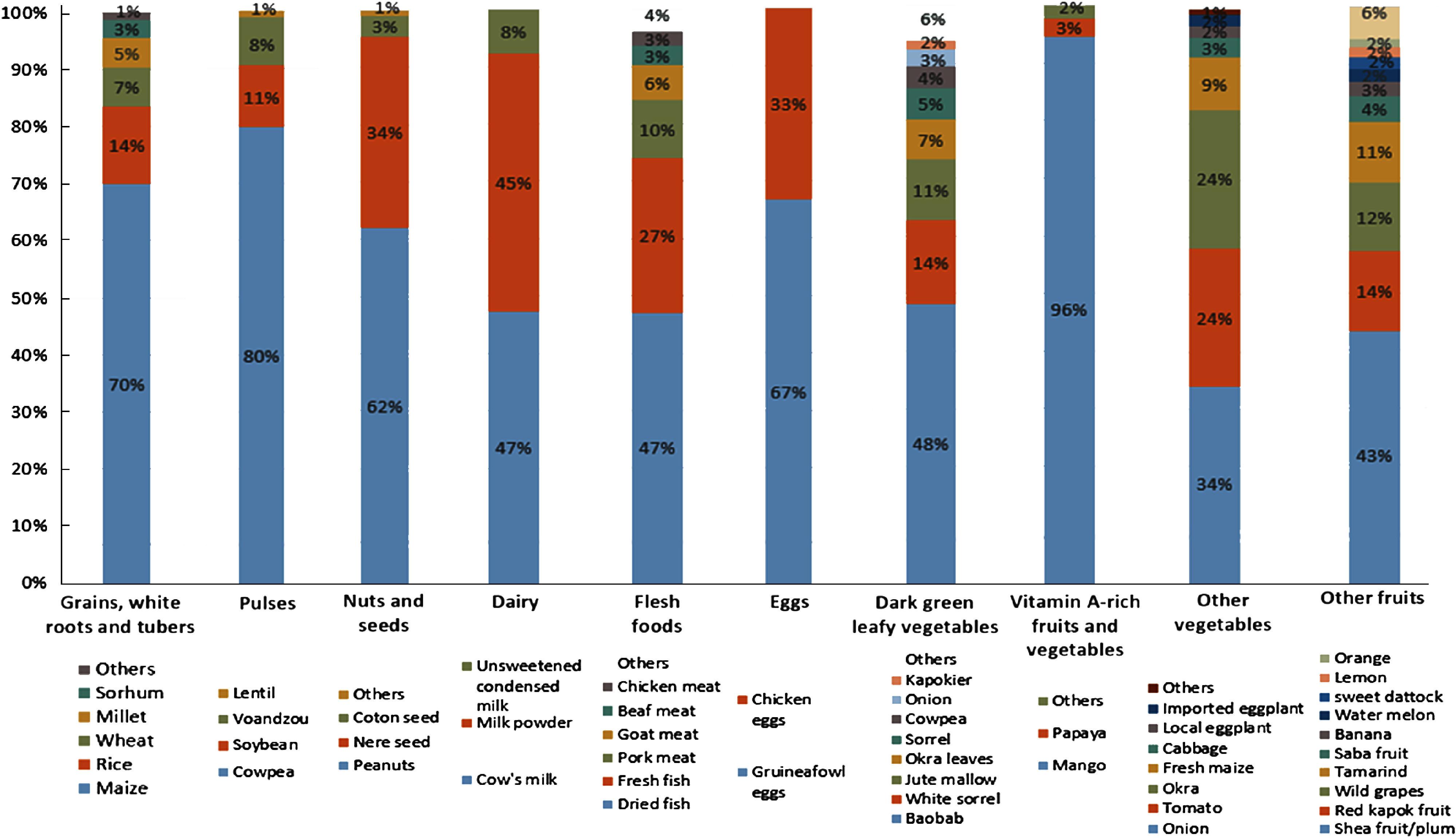
Contribution of food items to each food group consumed by women, averaged over the year (October 2017–September 2018). Foods items were included if they constituted at least 1 % of food items consumed within the food group

Most food groups consumed by the women were represented by only one or two food items (Fig. 2). Over the year, we observed that 70 % of the starchy staples consumed was maize, 80 % of the beans and peas were cowpeas and 96 % of the vitamin A-rich fruits and vegetables were mangoes. The main contributors to the ‘nuts and seeds’ group were peanuts (45 %) and néré seeds (33 %), and the main contributors to the ‘flesh foods’ group were dried fish (47 %) and fresh fish (27 %). Only the ‘leafy vegetables’, ‘other fruits’ and ‘others vegetables’ groups presented some internal variety, though yet again only a few foods predominated. Half of ‘leafy vegetables’ were baobab leaves, 15 % were white sorrel and 11 % were horn-fruited jute, while 43 % of ‘other fruits’ were shea plums, 14 % were red kapok fruits and 12 % were African grapes. Among the vegetables consumed, 34 % were onions, 25 % were okra and 24 % were tomatoes.

This lack of intra-group variety mainly explained the seasonality of dietary diversity and food group consumption observed among women (see online Supplemental Table 2). The higher proportion of women consuming ‘vitamin A-rich fruit and vegetables’ was observed from March to May during mango season. The higher consumption of ‘other fruits’ observed in May was explained by the abundance of wild grapes, while that in June–July was due to ample shea plums. The other vegetables consumed by women mostly came from horticultural crops from January to May, in particular onions and tomatoes, and from rainy crops from August to December, in particular fresh maize and okra. The main contributors to the ‘nuts and seeds’ group were peanuts from September to March (harvest period) and in June – July (sowing period), and néré seeds from October to December and from March to June.

### Supply sources of food groups

**Fig. 3:**
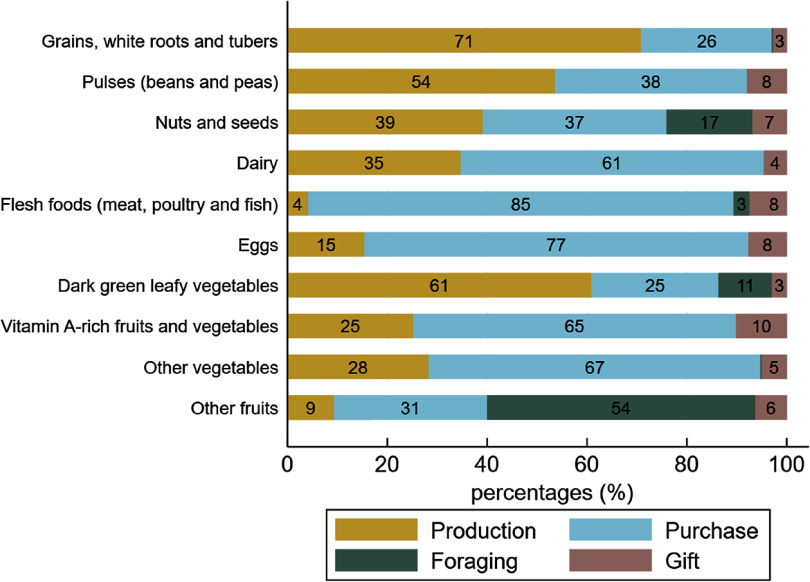
Share of food consumed provided by production, purchase, foraging and gifts for each food group. Figures were rounded to the nearest whole number

Within each food group, food items consumed by the women were provided by a primary supply source (Fig. 3 and see online Supplemental Table 2). We observed that 71 % of foods from the ‘starchy staple’ group were supplied by on-farm production over the year. Likewise, 61 % of leafy vegetables consumed by the women came from on-farm production, though horn-fruited jute was foraged. The foods consumed from the ‘beans and peas’ group were also primarily produced on-farm. In contrast, 85 % of the ‘flesh foods’ (dried and fresh fish) and 67 % of vegetables (onions and tomatoes) were purchased. Eggs, vitamin A-rich fruits and vegetables (mostly mangoes) and dairy products were also mainly purchased (77 %, 65 % and 61 % of items, respectively). In this latter group, cow milk was mostly produced but other dairy products like milk powder and unsweetened condensed milk were purchased. Within the ‘other fruits’ group foraging was predominant (54 %), followed by purchases (30 %). Shea fruit, red kapok fruit, wild grapes, tamarind, sweet datok and similar fruits were gathered, while orchard fruits such as bananas, watermelon, lemons and oranges were purchased at markets (30 %). Foods from the ‘nuts and seeds’ group presented mixed supply sources. The share of foods given as gifts was extremely low.

### Seasonal variations in food supply sources

**Fig. 4:**
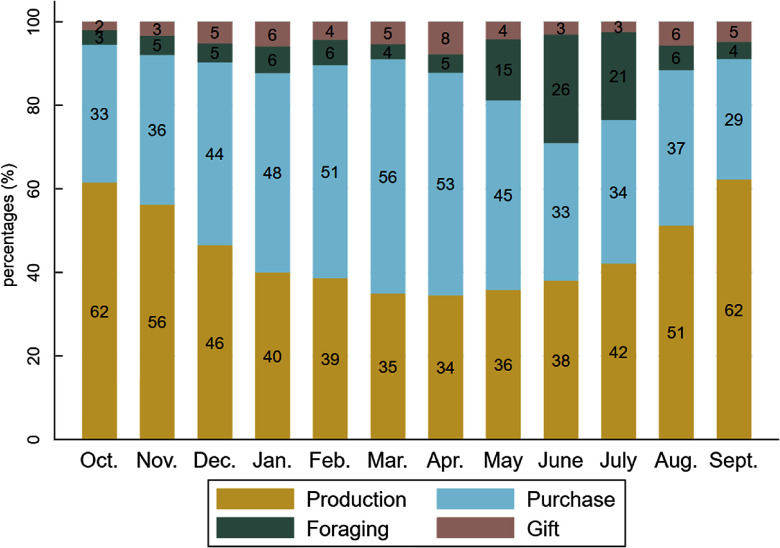
Share of food consumed provided by production, purchases, foraging and gift for each month. Figures were rounded to the nearest whole number

The shares of food supply sources varied according to seasons, following the pattern of agricultural cycles (Fig. 4). The consumption of food items from own-farm production (cereals, cowpeas, peanuts, fresh maize, baobab leaves) was highest in October–November (62 % and 56 %, respectively), then gradually declined until April (34 %) and slowly rose again to its initial level in September (62 %). The reduction of production as a food supply source observed between December and April was offset in part by increased food purchases, likely due to the availability of horticultural crops (onions and tomatoes) at markets, with a peak in March (56 %) during the mango season, and additionally by increased foraging of foods, notably the shea plums and wild grapes gathered from May to July. Gifts of food increased in April (mainly mangoes) and in August–September (peanuts), at the beginning of the rainy crop harvest.

### Factors associated with the seasonal variations in dietary diversity

**Fig. 5:**
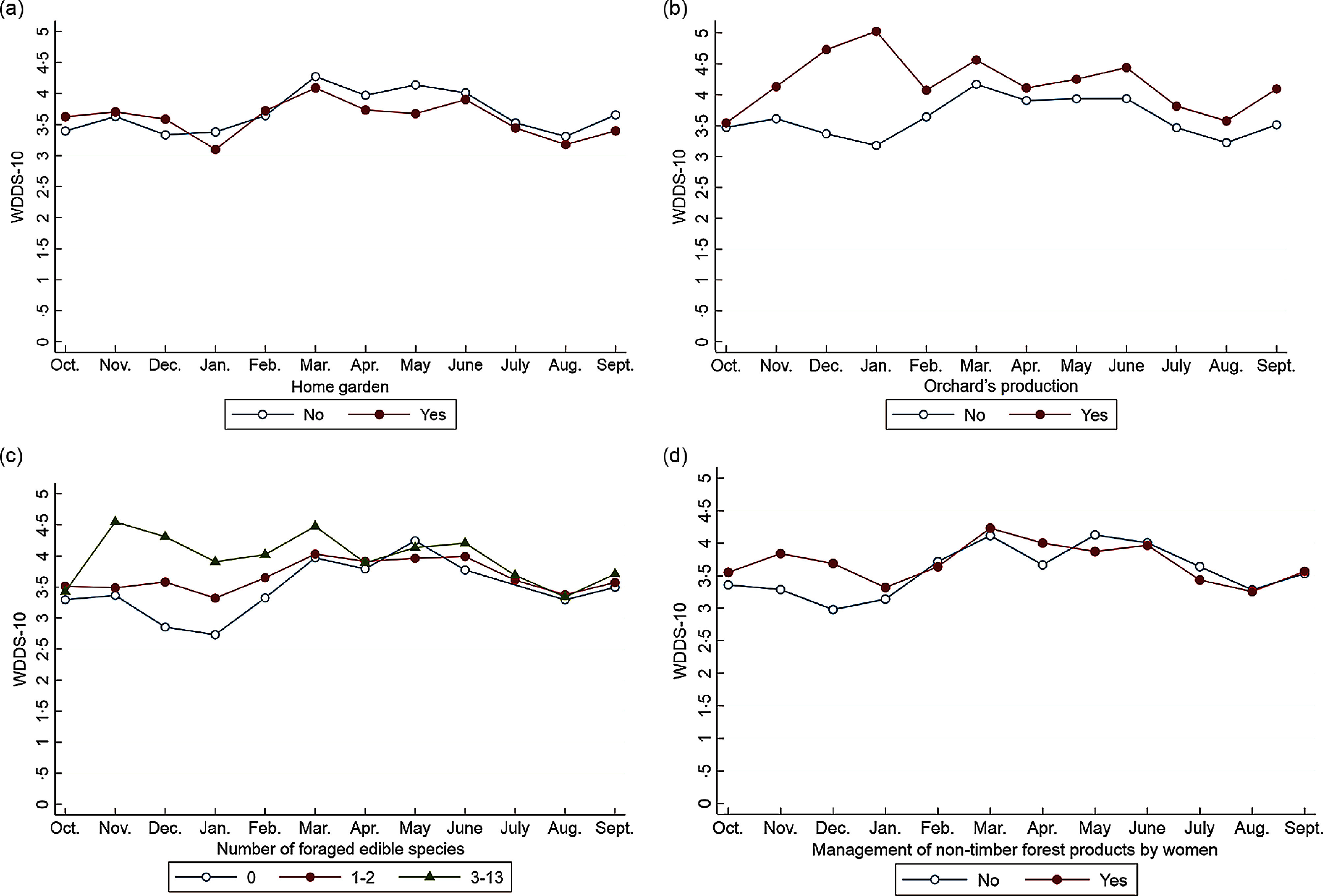
Seasonal variation in women’s dietary diversity according to farms’ and women’s characteristics, after adjustment for covariates (multivariate mixed model)

Over the year, WDD was positively associated with women’s literacy level (*P* < 0·1) and off-farm activities (*P* < 0·05) (Table 2). No association was found with the involvement of women in agricultural production on their own (whether a plot or a home garden). The WDDS-10 was also associated with farm characteristics such as the number of animal species and the number of plough oxen owned. Orchard production, foraging several species for food and the practice of more than two off-farm activities at the farm level were associated with a higher WDDS-10. Inversely, cotton production within farms was associated with a lower WDD-10 (*P* < 0·01) over the year. The interaction terms were significant for several variables, including the management of non-timber forest products at the women’s level, orchard production and foraging several species for food at the farm level, suggesting that the changes in WDDS-10 over the year were different across modalities for these variables. Stratified analyses show that these characteristics provided benefits to women, in terms of dietary diversity, only at certain times of the year (Fig. 5). Managing non-timber forest products, producing orchards and foraging a higher number of food species were associated with higher WDD from November to January/February in particular. Surprisingly, the WDDS-10 was lower among women living on farms with home gardens in January and from March to May.

**Table 2:** Associations between women’s dietary diversity scores (WDDS-10) and women’s and farms’ characteristics

	*n*	Coefficient		Interaction factor*months (*P*-value)
Months (reference: October 2017)	300			
November 2017	299	0·16	****	
December 2017	299	−0·05		
January 2018	299	−0·22	**	
February 2018	288	0·19	*	
March 2018	291	0·71	***	
April 2018	283	0·39	***	
May 2018	289	0·46	***	
June 2018	291	0·48	***	
July 2018	292	0·01		
August 2018	290	−0·23	**	
September 2018	289	0·06		
Women’s characteristics				
Women’s age (reference: < 30 years)	129			0·9
30 to < 40 years	104	0·01		
40 years and more	67	−0·12		
Women’s literacy (yes)	67	0·17	****	0·25
Women’s resources				
Manage plots (yes)	225	0·01		0·62
Manage home garden(yes)	99	−0·04		0·08
Manage non-timber forest products (NTFP) (yes)	190	0·06		0·000
Off-farm activities (yes)	229	0·21	*	0·11
Household characteristics				
Household size (terciles, reference: 1–5 people)	127			0·45
6–8 people	95	−0·10		
9–61 people	78	0·07		
Number of kinds of goods possessed by the household (radio, moto, moto-trailer, reference: 0)	54			0·6
1	93	−0·06		
2	125	0·05		
3	28	−0·05		
Farm characteristics				
Farm size (terciles in ha) (reference: <4 ha)	98			0·15
4–8	104	0·02		
9–75	98	0·04		
Production of cotton (yes)	202	−0·24	**	0·2
Horticultural plot (yes)	26	0·14		0·05
Home garden (yes)	124	−0·09		0·000
Orchard production (yes, every month)		0·28	***	0·002
Number of foraged edible species (terciles, reference: 0, collected monthly)				0·000
1–2		0·31	***	
3–13		0·47	***	
Number of animal species (quartile, reference: 0, collected monthly)				0·9
1–2		0·42	**	
3		0·37	*	
4–6		0·35	*	
Number of plough oxen (reference: 0)				0·25
1–2		0·08		
3–30		0·28	**	
Number of off-farm income sources (reference: 0, collected monthly)				0·08
1		0·11		
2 or more		0·16	*	
Constant		2·60	***	
Sample size		3510		

Values shown in column 2 are coefficients derived from a multivariate mixed model (with individuals and villages as nested random-effect intercepts), with WDDS as dependent variable.

Values in column 3 are *P*-values for interaction term = months × factor.

* *P* < 0·05.

** *P* < 0·01.

*** *P* < 0·001.

**** *P* < 0·10.

## Discussion

Our study found very low levels of dietary diversity among women of reproductive age in the Tuy province, Burkina Faso. As already reported in this country^(6,16,19,28)^, this is explained by the daily consumption of *tô*, the traditional family dish^(28,29)^, a porridge made of maize flour served with a sauce cooked with leafy vegetables and occasionally garnished with other vegetables (tomato, onion, okra), nuts and seeds (peanuts and néré seed) or fish. Thus, dietary diversity primarily relies on the sauce cooked with the *tô* and its variation from 1 d to another.

Furthermore, women’s diets were based on a narrow range of food items within food groups. In rural Burkina Faso, the food system is ‘traditional’, almost autarkic, and primarily characterised by the consumption of non-processed foods which are locally produced on-farm, foraged or purchased on informal markets^(30)^. In our study area, agricultural production is mainly based on the maize–cotton association, which offers little production diversity and therefore little food availability in local markets. Financial constraints and cultural norms are also recognized as major limiting factors in the consumption of expensive foods, including animal products and fruits^(31)^. Lastly, most perishable foods are consumed shortly after harvest because sun drying is the sole local conservation method. These factors may explain why the consumption of all food groups, except ‘dark green leafy vegetables’, ‘other vegetables’ and ‘other fruits’, was driven by only one or two food items from each. When a predominant food item is no longer accessible, the food group to which it belongs essentially vanishes from women’s diets, thus reducing the chance nutrient needs are met. Leveraging actions to enlarge the repertoire of food items women consume is likely to increase their dietary diversity and enable their nutrient needs to be met.

WDD was also seasonal. Both, the MDD-W and the WDDS-10 were at their highest levels between March and June, due to a higher availability and consumption of the ‘green leafy vegetables’ (mainly baobab leaves), ‘vitamin A-rich fruits and vegetables’ (mainly mangoes), ‘other fruits’ (shea plums and African grapes) and ‘other vegetables’ food groups (onions and tomatoes). Likewise, food sources’ supply was seasonal. By triangulating our data on food consumption and supply over the year, we identified three key periods: (i) during the harvest of rainy crops (August–January), more women consumed peanuts, cowpeas and some leafy vegetables (baobab leaves and white sorrel) or vegetables (fresh maize or okra), and the foods they consumed mainly came from on-farm production; (ii) during the harvest of horticultural crops (February–April), more women consumed mangoes and vegetables (onions and tomatoes); these foods were mainly purchased, thus compensating for the reduction in consumption of home-grown foods; and (iii) from May to July, more women consumed foraged ‘other fruits’ (shea plums), néré seeds and ‘leafy vegetables’ (horn-fruited jute), allowing a reduction in purchases.

As in similar contexts^(6,16,32)^, our data confirmed that the cereal shortage period overlaps with the rainy season, which increases the availability of horticultural and freely foraged foods and results in higher dietary diversity. Research work on dietary diversity should therefore take into account this breakdown into three key periods—harvest of rainfed crops, harvest of horticultural products, availability of free foods for foraging—rather than resorting to the overly simplistic dichotomies of ‘wet’ and ‘dry’ seasons or ‘lean’ and ‘harvest’ seasons.

Our results also suggest that farm production, market purchases and foraging are seasonal and complementary, and that all three sources play important interrelated roles in dietary diversity over the course of the year^(14,16,33–35)^. A better understanding of the underlying strategies, decision-making processes and constraints of households and women regarding food supply is essential to design interventions relevant to increased access to diversified foods. Moreover, we highlighted the non-equivalence between food supply sources—production, purchases and foraging—which give access to different foods at different times of the year. On-farm production accounted for a significant part of staple and basic foods, such as maize, baobab leaves, white sorrel, peanuts and okra, which formed the basis of food consumption. Purchased foods were mainly processed (e.g. pasta, bread, milk powder), but also included vegetables whose production requires significant land and irrigation resources, as well as flesh foods, because farm animals are preferably sold rather than consumed (with the exception of chicken). Foraging was the major source of ‘other fruits’ and néré seeds, which were less likely to be purchased, probably because they are considered supplementary foods. However, women only foraged a very limited number of food items, in our case shea and néré trees which represent a valuable source of income. We assume that foraging is practiced by women who either own or have access rights to trees, but it does not appear to be a livelihood strategy that enables farmers to access food for free. Foraging free wild food could certainly be promoted and extended to further locally available food products—for example, seeds, tubers and leafy vegetables—to allow a reduction in the production or purchase of these food groups, thus saving time, land and money. In providing nutritious foods and a source of income, natural resources are recognized as key determinants of household food security and resilience, especially for more economically vulnerable and during cereal shortage periods^(34,36,37,38)^.

Multivariate analyses showed that WDD-10 was positively associated with factors that permitted food availability and both financial and physical access to a larger range of foods at different time of the year. In line with other studies^(7,8,39)^, we highlighted that factors related to agricultural biodiversity—at similar farm size—such as orchard production, number of foraged edible species, the number of animal species and plough oxen owned by the household were associated with greater WDD-10 over the year. Household off-farm income was associated with higher WDDS-10 throughout the year, and in particular with a higher proportion of women consuming ‘flesh foods’, ‘other vegetables’ and mangoes bought on markets, while women’s off-farm income was associated with a higher proportion of women consuming foods from the ‘nuts and seeds’ group (results not shown), which suggests choices related to food purchases may be gendered. As previously observed by a study in the same region^(5)^, the production of cotton was negatively associated with the WDDS-10 over the year. Since cotton-related income is usually managed by the eldest males within households^(40,41)^, women may not directly benefit from it. This study have also documented that women who managed cotton crops themselves had a higher dietary diversity^(5)^, probably because they elect to spend more of their income on food purchases^(42–45)^. Interestingly, some associations between the WDDS-10 and women/farm characteristics were not constant over the year and instead followed seasonal harvests^(6)^. Factors such as horticultural plots, orchard production, management of non-timber forest product by women, the number of foraged edible species and the number of off-farm income sources were most beneficial for WDD from November to February—March, i.e. when most food consumed came from on-farm production. The advantages provided by these activities in terms of dietary diversity gradually declined with time, and disappeared when cereal stocks declined and fruits and vegetables (e.g. mangoes) became available at markets or in the wild. In contrast, women living in farms with home gardens had lower WDDS-10 than the other women in January and in March–May. We assume that home gardens are cultivated in small areas around the land concession as a livelihood strategy, to ensure a minimum of vegetables and leafy vegetables to garnish sauces in the absence of horticultural plots or sufficient financial resources to access them on the market.

To conclude, we highlighted that the MDD-W varied significantly (10 % to 39 % women) across different months and that agricultural biodiversity, income/market and natural resources are all potential contributors to WDD since they are seasonal and complementary over the year. This reinforces the need to use longitudinal studies to get a full picture of dietary diversity and its supply sources in similar contexts, or at least to conduct studies during months when the MDD-W is low to effectively identify the most vulnerable people. But above all, this means that integrated and complementary interventions and policies should be pursued in order to ensure food and nutrition security for all and at all times^(46)^. While securing on-farm production for the provision of staple foods remains crucial for vulnerable farms and individuals, several underutilised food groups and foods could be promoted. Animal products, in particular eggs, and rainy crops, such as cowpeas or *voandzou* (Bambara groundnut), which are little consumed and mainly purchased, could be promoted as cultivable and valuable foods, to be both consumed on-farm and sold for income. Traditional local plants and vegetables may also be further promoted, as in other African countries where higher consumption of these products was related to increased production^(10)^. Enhancing foraging appears as another way to improve dietary diversity, especially during the cereal shortage period. However, further research is needed on the availability of these resources and vulnerable women’s access to them, as well as on sustainable harvesting levels in a context of growing population and reduction of natural resources. Lastly, post-harvest management and conservation methods (drying, boiling, smoking, fermentation, etc.) should be further developed in this context to stabilise, or at least extend, the consumption of perishable foods throughout the year. Particularly, mangoes, which are largely available and consumed only 2 months in the year, could be dried to limit waste and hence become an affordable vitamin A-rich fruit over a longer period, in addition to providing extra income. A comprehensive appraisal of the local food system is required to assess how feasible these interventions would be in this setting.

## Supplementary material

For supplementary material accompanying this paper visit https://doi.org/10.1017/S1368980021004171. click here to view supplementary material
