# Supplementary material for: Seasonal variability of women’s dietary diversity and food supply: a cohort study in rural Burkina Faso
Source: Public Health Nutr. 2021 Oct 4;25(9):2475–87. doi: 10.1017/S1368980021004171 (PMC9991763; doi:10.1017/S1368980021004171)
Supplement: Supplementary file 1 [file S1368980021004171sup001.docx]

### Supplementary material

Supplementary table 1: Monthly variations in food groups consumption (October 2017 – September 2018)

Adjusted percentages for each food group are presented.

+ P<0.10, * P<0.05, ** P<0.01, *** P<0,001 correspond to significant differences between the food groups consumption at the given month compared with October. These p-values are derived from logit three-level mixed models (with individuals and villages as nested random-effects intercepts).

Supplementary table 2: Monthly variations in food groups and food items consumption (October 2017 – September 2018)

|  | Oct. 17 | Nov. 17 | Dec. 17 | Jan. 18 | Feb. 18 | Mar. 18 | Apr. 18 | May 18 | June 18 | July. 18 | Aug. 18 | Sept. 18 | Mean |
| --- | --- | --- | --- | --- | --- | --- | --- | --- | --- | --- | --- | --- | --- |
| **Starchy staples** | **99%** | **99%** | **100%** | **100%** | **99%** | **100%** | **100%** | **100%** | **99%** | **100%** | **99%** | **100%** | **100%** |
| Maize | 97% | 96% | 92% | 93% | 91% | 92% | 92% | 90% | 91% | 93% | 91% | 94% |  |
| Wheat | 9% | 16% | 15% | 12% | 10% | 9% | 7% | 3% | 6% | 6% | 5% | 5% |  |
| Millet | 3% | 9% | 10% | 7% | 9% | 6% | 7% | 19% | 3% | 3% | 3% | 3% |  |
| Rice | 14% | 17% | 20% | 18% | 23% | 18% | 22% | 16% | 17% | 16% | 19% | 17% |  |
| Sorghum | 2% | 1% | 3% | 2% | 5% | 5% | 4% | 7% | 6% | 7% | 5% | 4% |  |
| **Beans and peas** | **14%** | **13%** | **13%** | **8%^+^** | **9%** | **9%** | **7%** | **3%**** | **5%***** | **8%*** | **14%** | **16%** | **10%** |
| Cowpea | 12% | 12% | 11% | 8% | 7% | 6% | 4% | 3% | 4% | 5% | 13% | 15% |  |
| Soybean | 0% | 1% | 1% | 2% | 1% | 3% | 3% | 0% | 1% | 0% | 0% | 0% |  |
| Voandzou | 2% | 0% | 1% | 0% | 1% | 0% | 1% | 0% | 1% | 1% | 0% | 0% |  |
| Lentil | 0% | 0% | 0% | 1% | 0% | 0% | 0% | 0% | 0% | 0% | 0% | 0% |  |
| **Nuts and seeds** | **56%** | **57%** | **51%** | **42%^+^** | **37%***** | **46%** | **39%*** | **33%**** | **46%** | **45%** | **24%**** | **39%** | **47%** |
| Peanuts (snack) | 36% | 43% | 35% | 29% | 19% | 21% | 14% | 12% | 24% | 23% | 11% | 30% |  |
| Peanuts (paste) | 11% | 14% | 14% | 10% | 10% | 11% | 11% | 13% | 7% | 11% | 4% | 5% |  |
| Nere seed | 22% | 18% | 17% | 12% | 16% | 19% | 22% | 17% | 22% | 11% | 11% | 14% |  |
| Cotton seed | 0% | 0% | 2% | 2% | 2% | 3% | 4% | 5% | 2% | 0% | 0% | 0% |  |
| **Dairy** | **7%** | **5%** | **7%** | **3%** | **3%** | **3%** | **3%** | **1%**** | **4%** | **5%** | **6%** | **3%*** | **4%** |
| Cow's milk | 3% | 3% | 2% | 2% | 3% | 2% | 2% | 0% | 1% | 1% | 2% | 1% |  |
| Milk powder | 4% | 3% | 2% | 2% | 1% | 0% | 0% | 0% | 3% | 3% | 3% | 2% |  |
| Unsweetened condensed milk | 0% | 0% | 1% | 1% | 0% | 0% | 0% | 0% | 0% | 0% | 0% | 0% |  |
| **Flesh foods** | **41%** | **45%** | **38%** | **43%** | **30%** | **37%** | **40%** | **54%*** | **35%** | **38%** | **28%** | **28%*** | **40%** |
| Dried fish | 18% | 25% | 27% | 25% | 11% | 17% | 17% | 27% | 18% | 16% | 10% | 11% |  |
| Fresh fish | 11% | 10% | 10% | 12% | 11% | 10% | 13% | 11% | 9% | 10% | 11% | 15% |  |
| Pork | 3% | 4% | 3% | 7% | 7% | 3% | 8% | 9% | 6% | 4% | 2% | 2% |  |
| Goat | 3% | 3% | 2% | 1% | 2% | 1% | 5% | 3% | 4% | 3% | 3% | 1% |  |
| Beef | 1% | 2% | 2% | 2% | 3% | 1% | 1% | 3% | 1% | 1% | 2% | 0% |  |
| Chicken | 1% | 2% | 2% | 1% | 2% | 2% | 3% | 0% | 1% | 1% | 1% | 0% |  |
| Mutton | 3% | 3% | 2% | 1% | 2% | 1% | 5% | 3% | 4% | 3% | 3% | 1% |  |
| **Eggs** | **0%** | **1%** | **1%** | **0%** | **0%** | **0%** | **0%** | **0%** | **1%** | **0%** | **0%** | **0%** | **0%** |
| Guinea fowl eggs | 0% | 1% | 0% | 0% | 0% | 0% | 0% | 0% | 1% | 0% | 0% | 0% |  |
| Chicken eggs | **0%** | **0%** | **1%** | **0%** | **0%** | **0%** | **0%** | **0%** | **0%** | **0%** | **0%** | **0%** |  |
| **Vit.A-rich green leafy vegetables** | **74%** | **70%** | **49%**** | **40%**** | **81%** | **75%** | **81%** | **88%***** | **90%***** | **97%***** | **78%** | **70%** | **75%** |
| Baobab leaf | 47% | 48% | 27% | 18% | 55% | 54% | 63% | 75% | 68% | 59% | 25% | 26% |  |
| White sorrel | 23% | 15% | 7% | 3% | 14% | 16% | 15% | 10% | 10% | 16% | 19% | 20% |  |
| Jute mallow | 4% | 16% | 5% | 4% | 8% | 2% | 4% | 3% | 21% | 40% | 17% | 9% |  |
| Okra leaf | 7% | 9% | 9% | 4% | 6% | 3% | 2% | 2% | 0% | 5% | 18% | 16% |  |
| Sorrel | 7% | 5% | 3% | 2% | 6% | 4% | 4% | 2% | 8% | 7% | 8% | 7% |  |
| Cowpea leaf | 3% | 1% | 1% | 2% | 3% | 6% | 1% | 2% | 1% | 5% | 10% | 8% |  |
| Onion leaf | 4% | 7% | 5% | 6% | 5% | 1% | 0% | 1% | 2% | 0% | 0% | 1% |  |
| Kapok leaf | 0% | 1% | 3% | 5% | 6% | 3% | 2% | 1% | 0% | 0% | 0% | 0% |  |
| **Vit.A-rich vegetables and fruits** | **1%** | **2%** | **2%** | **0%** | **5%** | **57%***** | **44%***** | **34%***** | **9%**** | **0%** | **1%** | **0%** | **15%** |
| Mango | 0% | 0% | 0% | 0% | 5% | 59% | 44% | 29% | 9% | 0% | 0% | 0% |  |
| Papaya | 0% | 1% | 2% | 1% | 0% | 0% | 0% | 0% | 0% | 0% | 0% | 0% |  |
| **Other vegetables** | **40%** | **38%** | **43%** | **58%**** | **74%***** | **74%**** | **65%**** | **54%*** | **36%** | **27%** | **73%***** | **93%***** | **53%** |
| Onion | 12% | 16% | 25% | 48% | 55% | 61% | 56% | 43% | 26% | 23% | 22% | 17% |  |
| Tomato | 21% | 14% | 27% | 47% | 51% | 44% | 23% | 11% | 2% | 3% | 12% | 33% |  |
| Okra | 20% | 20% | 12% | 6% | 25% | 34% | 23% | 11% | 8% | 8% | 55% | 66% |  |
| Fresh maize | 0% | 0% | 0% | 0% | 0% | 0% | 0% | 0% | 0% | 0% | 39% | 71% |  |
| Cabbage | 1% | 4% | 6% | 9% | 7% | 4% | 5% | 1% | 0% | 0% | 0% | 2% |  |
| Local eggplant | 1% | 1% | 2% | 1% | 1% | 0% | 0% | 0% | 0% | 0% | 10% | 8% |  |
| Imported eggplant | 0% | 1% | 2% | 1% | 3% | 1% | 2% | 1% | 2% | 1% | 4% | 3% |  |
| **Other fruits** | **5%** | **13%*** | **23%**** | **17%*** | **17%**** | **14%*** | **12%** | **44%***** | **83%***** | **42%***** | **2%*** | **2%** | **26%** |
| Shea plum | 0% | 0% | 0% | 0% | 0% | 0% | 0% | 5% | 81% | 47% | 1% | 0% |  |
| Red kapok fruit | 0% | 2% | 8% | 6% | 7% | 7% | 7% | 4% | 2% | 0% | 0% | 0% |  |
| Wild grape | 0% | 0% | 0% | 0% | 0% | 0% | 0% | 37% | 1% | 0% | 0% | 0% |  |
| Tamarind | 1% | 6% | 5% | 8% | 3% | 2% | 2% | 4% | 0% | 0% | 0% | 0% |  |
| Saba fruit | 0% | 0% | 0% | 0% | 0% | 0% | 0% | 4% | 9% | 0% | 0% | 0% |  |
| Banana | 1% | 1% | 2% | 1% | 0% | 0% | 0% | 0% | 0% | 0% | 0% | 1% |  |
| Water melon | 2% | 3% | 2% | 0% | 0% | 0% | 0% | 0% | 0% | 0% | 0% | 0% |  |
| Sweet dattock | 0% | 0% | 0% | 0% | 4% | 2% | 0% | 0% | 0% | 0% | 0% | 0% |  |
| Lemon | 1% | 2% | 1% | 0% | 0% | 0% | 0% | 0% | 0% | 0% | 0% | 0% |  |
| Orange | 0% | 2% | 2% | 0% | 0% | 0% | 0% | 0% | 0% | 0% | 0% | 0% |  |

Adjusted percentages for each food group are presented.

+ P<0.10, * P<0.05, ** P<0.01, *** P<0,001 correspond to significant differences between the food groups consumption at the given month compared with October. These p-values are derived from logit three-level mixed models (with individuals and villages as nested random-effects intercepts).

Supplementary figure 1: Source of food consumed within each food group

Foods items were included if they constituted at least 1% of food items consumed within the food group.
